# Survey Results of Honey Bee Colony Losses in Winter in China (2009–2021)

**DOI:** 10.3390/insects14060554

**Published:** 2023-06-14

**Authors:** Jiao Tang, Congcong Ji, Wei Shi, Songkun Su, Yunbo Xue, Jinshan Xu, Xiao Chen, Yazhou Zhao, Chao Chen

**Affiliations:** 1 State Key Laboratory of Resource Insects, Institute of Apicultural Research, Chinese Academy of Agricultural Sciences, Beijing 100193, China; 2 College of Animal Sciences (College of Bee Science), Fujian Agriculture and Forestry University, Fuzhou 350002, China; 3 Jilin Province Institute of Apicultural Science, Jilin 132000, China; 4 College of Life Sciences, Chongqing Normal University, Chongqing 401331, China

**Keywords:** colony winter losses, honey bee health, China, risk factor, *Apis mellifera*, *Apis cerana*

## Abstract

**Simple Summary:**

Honey bees are of great importance because of their roles in pollination and the supply of bee products. However, the number of honey bee colonies is declining worldwide, and these colony losses mainly occur in winter. Colony loss surveys have been regarded as an efficient measure to protect managed honey bees, as they help identify potential risk factors for colony loss. They may also make beekeepers pay more attention to overwinter beekeeping management and thus reduce colony losses. We conducted surveys on the overwinter mortality of managed honey bee colonies in China from 2009 to 2021. The aim of the present study was to evaluate the health status of honey bee colonies in China and describe the risk factors for winter colony losses. We reported that colony losses were low, with variations among years, provinces, species (*Apis mellifera* and *Apis cerana*), and types of apiaries. The results showed that the queen problems (queenless colonies or drone-laying queens), operation size, migration, migration×species interaction, and species significantly affected winter colony losses. Our study contributes to improving the health of managed honey bees and provides useful strategies for colony overwintering.

**Abstract:**

There is growing concern that massive loss of honey bees can cause serious negative effects on biodiversity and ecosystems. Surveys of colony losses have been performed worldwide to monitor the dynamic changes and health status of honey bee colonies. Here, we present the results of surveys regarding winter colony losses from 21 provinces in China from 2009 to 2021, with a total of 1,744,324 colonies managed by 13,704 beekeepers. The total colony losses were low (9.84%; 95% Confidence Interval (CI): 9.60–10.08%) but varied among years, provinces, and scales of apiaries. As little is known about the overwintering mortality of *Apis cerana*, in this study, we surveyed and compared the loss rates between *Apis mellifera* and *A. cerana* in China. We found colonies of *A. mellifera* suffered significantly lower losses than *A. cerana* in China. Larger apiaries resulted in higher losses in *A. mellifera*, whereas the opposite was observed in *A. cerana*. Furthermore, we used generalized linear mixed-effects models (GLMMs) to evaluate the effects of potential risk factors on winter colony losses and found that the operation size, species, migration, migration×species interaction, and queen problems were significantly related to the loss rates. New queens can increase their colony overwintering survival. Migratory beekeepers and large operations reported lower loss rates.

## 1. Introduction

As one of the major insect pollinators, honey bees are of vital significance to global food security and ecological balance [1,2]. However, pathogen invasion and pesticide abuse have led to a decline in honey bee colonies, resulting in severe threats to biodiversity, agriculture, and ecosystem [3,4,5]. Mounting evidence suggests that a decline in feral and managed honey bee colonies has occurred worldwide [6,7]. Consequently, colony losses have been surveyed across several continents in recent years [8,9,10,11]. Unexpectedly, the United States witnessed a sudden death of honey bee colonies in 2006, which raised great concern for colony losses. Since then, the overwintering mortality of honey bees has been investigated annually in the United States [12,13,14,15,16,17,18]. Soon after, many countries in Europe jointly surveyed winter colony losses in the efforts of Prevention of honey bee COlony LOSSes (COLOSS) [19,20,21,22,23,24]. Currently, colony losses are surveyed in many parts of the world, including Asia [25,26,27,28], Africa [29], the Americas [14,30,31,32,33,34,35], Europe [36,37,38], and Oceania [9,39], most of which present high loss rates. In China, continuous and systematic surveys on the winter mortality of managed honey bee colonies date back to 2009, even though a massive colony collapse has not been reported yet. To date, several colony loss surveys in China reported that the overall mortality of honey bees is maintained at a low level. Moreover, this loss rate is substantially influenced by risk factors such as queen problems, operation size, and the proportion of new queens [26,27,28].

Most areas of China lie in the northern temperate zone, which experiences cold winters. As honey bees cannot forage at low temperatures, overwintering is very important for honey bee colonies in temperate climates [40]. Colonies managed in northern China usually overwinter for about 5 months—from November to March. In Northeast China, where areas are of higher latitudes, the overwintering period is usually longer, extending by 10 to 30 days, and the minimum temperature during the winter period is lower. Some provinces in Central China, such as Anhui and Hubei, have a shorter wintering period (lasting for three months). Chinese beekeepers have adopted several management practices for the overwinter survival of honey bee colonies, including treatment against parasites (such as *Varroa destructor*), the construction of overwintering core colonies (strong colonies formed by merging colonies or supplementing capped brood) before winter, feeding sugar to the bees before winter, and keeping them warm during the winter. In China, honey bee colonies are mostly managed to provide bee products and pollination services, but a small part of the products are used for traditional Chinese medicines, such as propolis and bee venom [41,42]. Beekeeping is also considered an important industry in China for poverty alleviation and rural revitalization.

Two bee species exist in China—*Apis mellifera* and *Apis cerana*—and there is a long history of managed beekeeping of *A. cerana*, which is native to Asia [43]. In China, *A. cerana* was domesticated on a large scale more than 2000 years ago, and currently, there are approximately 2 million *A. cerana* colonies in the country [44]. *A. mellifera* was first introduced into China from Tsarist Russia in the late 1800s [45,46], and the first native *A. mellifera* subspecies was identified in China in 2016, as reported in our previous study [47]. *A. mellifera* and *A. cerana* exhibit some similarities and differences in their phenotypes, physiological characteristics, and biological habits [48,49,50]. For example, *A. cerana* is resistant to *V. destructor* and can forage under lower temperatures than *A. mellifera* [51,52]. Additionally, *A. cerana* outperforms *A. mellifera* with respect to the use of scattered nectar resources [51,52]. Recent studies on winter colony losses have focused on *A. mellifera*, and colony loss surveys regarding *A. cerana* are extremely rare worldwide. To our knowledge, this is the first national survey on the differences in winter colony losses between two different bee species—*A. mellifera* and *A. cerana*—in China.

Here, we report the results of a continued survey of winter colony losses from 2009 to 2021 and explore the possible factors that influence the overwintering mortality of honey bees using linear models. Specifically, we found significant differences in the loss rates of different honey bee species.

## 2. Materials and Methods

### 2.1. Survey Design and Data Validation

Survey questionnaires were designed in accordance with the COLOSS standardized questionnaire published by van der Zee et al., in 2013 [53] and modified for the apicultural context of China. The questionnaires were printed in Chinese, and all questions in the questionnaires were the same, regardless of the honey bee species. Questionnaires were shared with local beekeeping organizations as well as randomly distributed to local beekeepers by trained surveyors. These surveyors collected information using telephonic or face-to-face interviews and then submitted it using a website from the years 2009–2015 or by email correspondence.

Our survey questionnaire was divided into two parts. The first part focused on colony losses and included parts of the COLOSS questionnaire. These questions include the number of colonies which the beekeeper had on 1st October, the number of colonies lost between 1 October and 1 April, the number of the lost colonies without dead bees in the hive, and the number of the lost colonies with dead bees in cells. The number of colonies lost between 1 October and 1 April was regarded as the total number of colonies lost in this study. The second part of the questionnaire covered several kinds of questions on hive management practices, such as the proportion of wintered colonies with a new queen in the apiaries, the frequency of requeening in one year, the origin of the queen, the number of colonies that have unsolved queen problems (drone laying queens or no queen at all), the practices of migratory beekeeping, treatment practices against *V. destructor*, and renewal of honeycomb. Some of these operational factors were selected according to the COLOSS questionnaire and some additional options specific to Chinese apiculture were also included in our questionnaires. The loss data was collected from the October 1st of one year to April 1st of the next year.

All data were exported from the database and imported into a personal computer for data cleansing. First, the dataset was filtered to remove duplications and contradictory entries (number of losses > original colony number or number of overwintering colonies = 0). If the responses of one province for the entire study period were fewer than five, data were removed. The remaining data were used to calculate overall loss rates in the form of total losses, as recommended by van der Zee et al. [53]. The total loss is the total number of colonies that were lost divided by the total number of colonies in the sample before winter and is expressed as a percentage.

### 2.2. Statistical Analysis

Our final dataset included twelve years of data, part of which has been reported previously (2010–2017, [26,27,28]), while the remaining data were acquired for the present study. Apiaries were classified into three categories according to the actual situation of Chinese apiculture and our previous criteria [26]: hobby (1–50 colonies), side-line (51–200 colonies), and commercial (>200 colonies). Confidence intervals (CI) were calculated using an intercept-only generalized linear model (GLM) with a quasi-binomial distribution and logit link function using the standard methods [53]. The data and the transformed data are not normally distributed; therefore, they presented significant differences in terms of variance. Thus, the Kruskal–Wallis rank sum test using individual loss rates of beekeepers was conducted to explore the differences between subgroups of respondents, with FDR methods for adjustment of *p*-values for multiple comparisons (Dunn’s Multiple Comparison Test). Subgroups with less than ten data points were excluded from comparison. In addition, we used data from six provinces (Chongqing, Gansu, Guangdong, Guangxi, Jiangxi, and Zhejiang) to compare provincial losses across years. These provinces were selected as they contributed consistently to our survey each year, whereas other provinces lacked data for some years. The risk factors for winter colony losses were tested using generalized linear mixed models (GLMM) with a binomial distribution. The risk factors included the proportion of new queens, queen problems, migration, operation size, pollination, frequency of requeening, comb renewal, winter food, and treatment against *V. destructor*. We first tested the factors individually as fixed effects, with provinces, years, and beekeepers as random effects, and included all eight significant factors in the full model. Next, we tested the significance of the factors in the full model and deleted non-significant terms so that the simplified model could be achieved. The simplified model was further validated by comparing the BIC and AIC values of the models with all the possible combinations of the eight factors (2^8^ = 256 models) to ensure the effectiveness of our model selection. Finally, possible interaction terms were tested, and a final model with an interaction term was formed based on BIC values.

All statistical analyses were conducted using R statistical software (R Development Core Team, Vienna, Austria, 2022) (version 4.2.1) [54]. Adjustment of *p*-values for multiple comparisons for the Kruskal–Wallis rank sum test was conducted using the FSA package (version 0.9.3) [55]. GLMMs analysis was performed using the “lme4” package (version 1.1.30) [56]. Graphs were drawn using GraphPad Prism (version 8.3.1).

## 3. Results

### 3.1. Survey Sample

**Figure 1:**
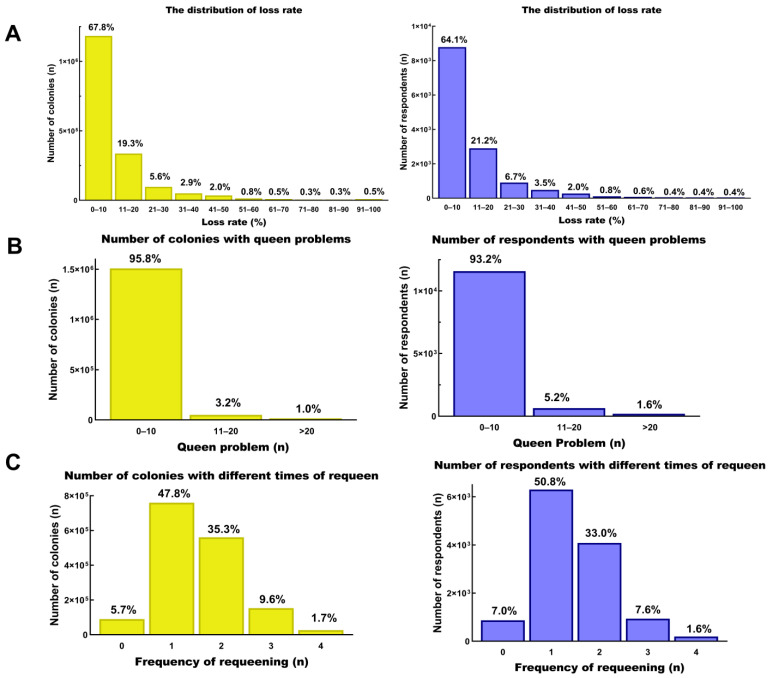
The distribution of colonies/respondents with loss rate interval, queen problems, and frequency of requeening. (**A**) The distribution of colonies/respondents with different loss rates. (**B**) The distribution of colonies/respondents with disabled queens. (**C**) The distribution of colonies/respondents with different frequency of requeening in one year.

In China, the obligatory registration of beekeepers is not yet widely implemented, which considerably hindered our ability to reach a wider community of beekeepers. In our survey, the majority of the data was collected during 2020–2021, which included a total of 266,173 colonies (15.3% of the total colonies surveyed in the present study) managed by 1739 respondents (12.7% of the total respondents) (Figure S1). The distribution of colony losses showed that the majority of the beekeepers (N = 8876, 64.1%) managing a total of 1,183,386 colonies (67.8%) had loss rates between 0 and 10% (Figure 1A). The distribution of the number of colonies with unsolvable queen problems showed that most of the participating beekeepers (N = 11,589, 93.2%) who managed 95.8% of the colonies (N = 1,508,793) had between 0 and 10 disabled queens (Figure 1B). Of the total respondents, 50.8% (N = 6301) reported replacing the queens once every year, with 47.8% (N= 760,339) of the colonies having the queens replaced once every year (Figure 1C). Treatment against *V. destructor* was performed in 99.4% of the *A. mellifera* colonies (N = 699,920) by most beekeepers (N = 4779, 99.1%), but almost all colonies (N = 376,698, 94.3%) of *A. cerana* were not treated (Figure S2A). The distribution of the percentage of comb renewal showed that 23.5% of the colonies (N = 369,184) had 31–40% new combs managed by most beekeepers (N = 2860, 23.5%) (Figure S2B). Furthermore, 36% of the beekeepers (N = 1593) migrated about 43% of the total colonies (N = 226,793), whereas 12% of the beekeepers (N = 534) who managed a total of 69,787 colonies (13%) provided pollination services. About 9% of the beekeepers (N = 404) stated that they migrated the colonies as well as provided pollination services, which corresponded to 11% of the total colonies (N = 56,431) (Figure S2C). As shown in Figure S2D, the number of commercial beekeepers (N = 969, 72.8%) who introduced queens from outside was apparently higher than that of hobby beekeepers (N = 899, 41.6%). Most of the participating beekeepers (N =8303, 66.8%) who managed 57.0% of the colonies (N = 897,380), belonging to sideline beekeepers, had between 0 and 10 disabled queens (Figure S3).

### 3.2. Winter Colony Losses in China (2009–2021)

In total, we received 13,704 valid responses from 21 provinces in China, leading to a sample size of 1,744,324 colonies. Of all the valid responses, 32% respondents reported that they did not suffer colony losses in winter. The calculated total losses for 12 consecutive years were relatively low (9.84%; 95% CI: 9.60–10.08%) (Table 1) compared with other regions of the world [9,11,17,19,20,21].

### 3.3. Annual Losses (2009–2021)

**Figure 2:**
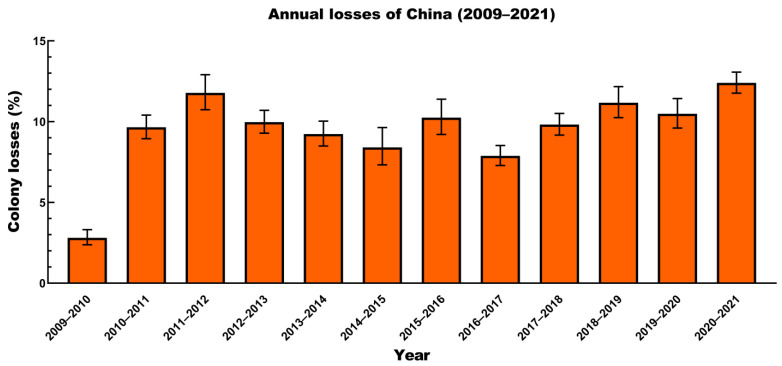
Winter colony losses (%, 95% CI) for different years (2009–2021).

The number of colonies for each year is shown in Table 1. As shown in Figure 2, the winter colony losses were differed by year, and the annual losses were generally low. The lowest annual loss (2.81%; 95% CI: 2.38–3.32%) in 2009–2010 was significantly different from the loss rate in other years (*p* < 0.00001), as validated by using Dunn’s multiple comparison test. The annual losses in 2020–2021 were significantly higher than those in the others (*p* < 0.0001). Again, this was validated using Dunn’s multiple comparison test.

### 3.4. Provincial Losses

**Figure 3:**
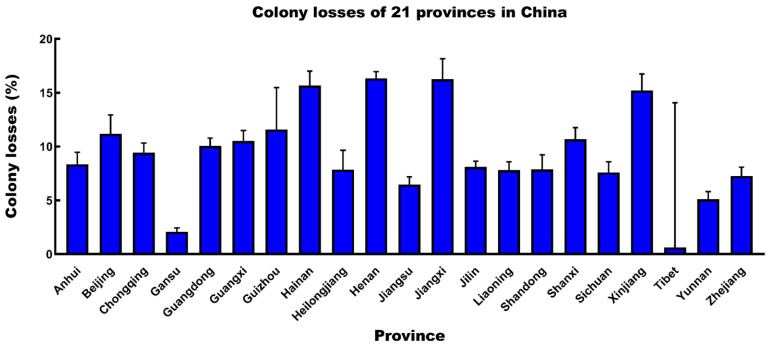
Winter colony losses (%, 95% CI) for different provinces in China.

The number of apiaries/colonies and the total losses in all surveyed provinces were calculated during 2009–2021, and notable variations were observed among the 21 provinces in China (Table 2). The loss rates at the provincial level varied from 2.09% (95% CI: 1.80–2.44%) to 16.34% (95% CI: 15.74–16.96%) (Figure 3). The minimum winter loss occurred in Gansu, which was significantly different from that in all other provinces (*p* < 0.00001). Henan reported the highest colony losses and was significantly different from the loss rates of other provinces (*p* < 0.005), except for Guizhou (*p* = 1). The significant differences were shown by the Dunn’s multiple comparison test, using individual loss rates.

To assess the differences between provincial colony losses, we selected six representative beekeeping provinces and analyzed the differences in annual losses of these provinces during 2017–2021. These provinces include Chongqing, Gansu, Guangdong, Guangxi, Zhejiang, and Jiangxi. Significant differences in annual colony losses in the six provinces were tested using Dunn’s multiple comparison test. The results showed that colony losses in Gansu were maintained at a low level between 2017 and 2021, which was significantly lower than those of the other provinces in 2019–2020 (1.08%; 95% CI: 0.65–1.78%) and 2020–2021 (2.34%; 95% CI: 1.66–3.31%) (*p* < 0.00001) (Table S1). The highest annual loss in Gansu was observed in 2018–2019 (3.31%; 95% CI: 2.41–4.52%) and was significantly different from other years (*p* < 0.005). In 2017–2018, the losses reported in Zhejiang (*p* < 0.005), Jiangxi (*p* < 0.0001), and Gansu (*p* < 0.005) were significantly different from those in the other provinces. Similarly, significant differences were observed involving Gansu (*p* < 0.05), Jiangxi (*p* < 0.00001), and Zhejiang (*p* < 0.05) in 2018–2019 (Table S1).

### 3.5. Operation Sizes and Loss Rates

**Figure 4:**
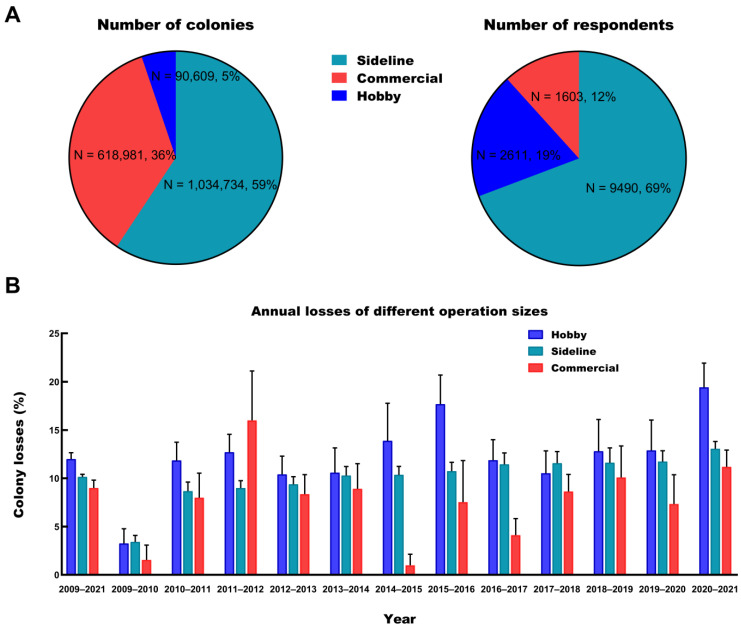
Comparison of the proportion and mortality of different apiaries. (**A**) The number of respondents/colonies by different types of apiaries. (**B**) Comparison of winter colony losses (%, 95% CI) by different types of apiaries for the years (2009–2021).

Our results demonstrated that most of the respondents (69%, N = 9490) were sideline beekeepers with a total of 1,034,734 (59%) colonies, and 36% of these sideline beekeepers reported no losses during winter (Figure 4A). Additionally, hobby beekeepers (19%, N = 2611) managed 5% of the colonies (N = 90,609), and 40% of them reported no losses. Commercial beekeepers (12%, N = 1603) managed 35% of the colonies (N = 618,981), and 36% of them reported no losses. The winter colony losses of hobby, sideline, and commercial beekeepers were 11.99% (95% CI: 11.36–12.65%), 10.15% (95% CI: 9.89–10.42%), and 8.99% (95% CI: 8.24–9.81%), respectively. Based on the colony loss data spanning 12 years, we concluded that the overwintering mortality of honey bees varied with the operation sizes, and larger-scale apiary owners reported lower mortality rates compared to small-scale apiary owners (Figure 4B). Only the loss rates of commercial beekeepers were significantly different from those of hobby/sideline beekeepers, according to Dunn’s multiple comparison test, which also considered the individual loss rates for the beekeepers (*p* < 0.00001). For the hobby beekeepers, the annual losses varied from 3.24% (95% CI: 2.19–4.77%) to 19.42% (95% CI: 17.15–21.92%). The lowest loss rate in 2009–2010 was significantly different from other annual losses, according to Dunn’s multiple comparison test (*p* < 0.00001). The mortality of sideline beekeepers was the highest in 2020–2021 (13.06%; 95% CI: 12.34–13.81%) and the lowest in 2009–2010 (3.41%; 95% CI: 2.85–4.08%). Both the highest and lowest values were significantly different from those of the other years, as determined by Dunn’s multiple comparison test (*p* < 0.001). For individual years, sideline beekeepers experienced significantly higher mortality than commercial and hobby beekeepers during 2009–2010 (*p* < 0.0001). The loss rates of commercial apiaries in 2014–2015 and 2020–2021 were significantly lower than those of the other two types of apiaries, according to Dunn’s multiple comparison test (*p* < 0.00001). There were significant differences in the loss rates among the three types of apiaries in 2015–2016 (*p* < 0.00001) and 2016–2017 (*p* < 0.005). Overall, with the exception of certain years, a trend of lower loss rates in larger operation sizes was observed.

### 3.6. Differences in Colony Losses between A. mellifera and A. cerana in China

**Figure 5:**
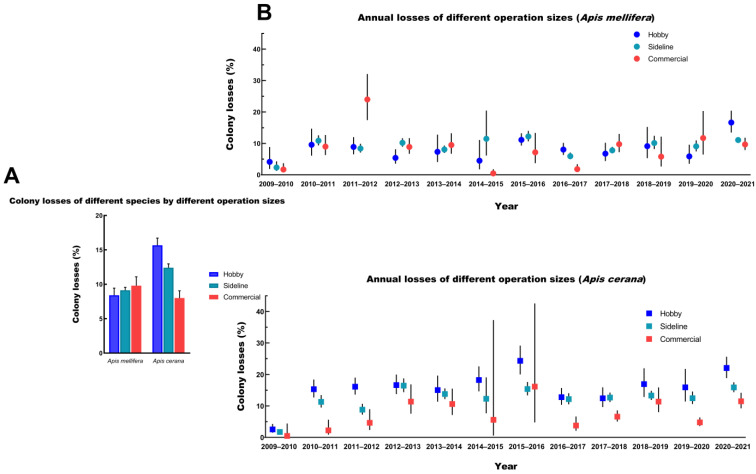
Variation of winter colony losses (%, 95% CI) by different species, types of apiaries, and years (**A**) Total winter colony losses (%, 95% CI) of different species in different types of apiaries. (**B**) *Apis mellifera* winter colony losses (%, 95% CI) by different types of apiaries for the years (2009–2021), upper panel. *Apis cerana* winter colony losses (%, 95% CI) by different types of apiaries for the years (2009–2021), lower panel.

Our dataset included both species and compared the results. The survey recorded a total of 847,587 and 478,905 colonies for *A. mellifera* and *A. cerana*, respectively. These were separately managed by 5711 beekeepers (*A. mellifera*) and 4253 beekeepers (*A. cerana*). The 12-year loss rates of *A. mellifera* (9.38%; 95% CI: 8.98–9.79%) were significantly lower than those of *A. cerana* (11.13%; 95% CI: 10.72–11.55%), as shown by Dunn’s test (*p* < 0.00001) (Table 3). We also found that, with the exception of 2009–2010, *A. mellifera* had significantly different annual losses from *A. cerana* (*p* < 0.05). Furthermore, the annual losses of *A. cerana* in 2009–2010 (1.56%; 95% CI: 1.10–2.21%) were significantly lower than those in the other years (*p* < 0.00001). For *A. mellifera*, the annual losses in 2009–2010 (*p* < 0.005) and in 2020–2021 (*p* < 0.0001) were significantly different from that in the other years, as tested by Dunn’s multiple comparison test. Our data showed that the two honey bee species had significant differences in winter colony losses among all types of apiaries, as tested by Dunn’s multiple comparison test (*p* < 0.00001). Meanwhile, the loss rates of *A. cerana* managed by different operation sizes were significantly different (*p* < 0.0005). However, for *A. mellifera*, only the loss rates of sideline beekeepers differed significantly from those of hobby and commercial beekeepers (*p* < 0.00001). When comparing the overall losses of the two honey bee species managed by different operation sizes, we found that the larger apiaries of *A. cerana* suffered lower losses, but the opposite trend was observed for *A. mellifera* (Figure 5A). When examined for each individual year, the pattern was generally consistent with the annual losses for *A. cerana*. In contrast, the annual losses of *A. mellifera* from apiaries of different sizes fluctuated wildly, with different years showing different patterns (Figure 5B). Migratory beekeepers who managed *A. cerana* (9.76%; 95% CI: 8.95–10.62%) had lower losses than their stationary counterparts (13.58%; 95% CI: 12.95–14.24%) (*p* = 8.64 × 10^−8^). Migrated *A. mellifera* colonies (8.42%; 95% CI: 7.91–8.96%) suffered a lower loss rate than migrated *A. cerana* (9.76%; 95% CI: 8.95–10.62%) (*p* = 1.23 × 10^−8^). *A. mellifera* colonies without pollination (11.09%; 95% CI: 10.29–11.94%) lost nearly more than twice the number of colonies for pollination (6.62%; 95% CI: 5.80–7.54%) (*p* = 0.0034).

### 3.7. Risk Factors Attributed to Winter Colony Losses

Our analysis of risk factors comprised a total of 6825 responses, and the potential risk factors included the proportion of new queens, frequency of requeening, honey bee species, comb renewal, treatment against *V. destructor*, queen problems, operation size, winter food, origin of queen, migration, pollination service, nectar source, and so on. GLMMs analysis was used to evaluate the influence of risk factors on winter colony losses. Consistent with previous studies, provinces, beekeepers, and years were added to the null generalized linear mixed model as random factors. After dropping non-significant terms, a simplified model with operation size, queen problems, migration, and species was formed (Table 4 and Supplementary Material S2). Among the four factors, species×migration and species×size showed signs of interaction. As a result, we added the interaction terms and, based on BIC values, reached a final model that includes one interaction term species×migration. In Section 3.5, it is clear that the operation size significantly affected the winter loss rate. Migration was also a significant influencing factor, as those who migrated their colonies had lower loss rates. In China, colonies of *A. mellifera* had lower losses than colonies of *A. cerana.* Young queens had a positive effect on colony survival. Our analysis revealed that treatment against *V. destructor* was not a significant variable for colony loss, which may be caused by insufficient data. It is well known that *V. destructor* control failure has a significant negative effect on colony loss.

## 4. Discussion

As one of the major beekeeping countries, China has a total of approximately 9 million managed colonies of two honey bee species, namely *A. mellifera* and *A. cerana*. Data from the Food and Agriculture Organization of the United Nations (FAO) show that the total number of honey bee colonies in China (2009–2021) is increasing [57]. In the present study, the number of responding colonies was 109,362,501, cumulatively covering 11.66% of the total honey bee colonies in China. The response rate in the present study was lower than that of other surveys, but this could be attributed to the difficulties in reaching beekeepers [9,15,16]. As Chinese beekeepers are not required to register, the availability of contact details of beekeepers is scarce. Moreover, only a small number of beekeepers were aware of the importance of the surveys and actively participated in our survey. Therefore, we acknowledge that convenient access to the questionnaires and providing rewards for participation could aid our investigations in the future.

To our knowledge, this is among the largest surveys of winter colony losses in China. A total of 13,704 Chinese beekeepers from 21 provinces reported a comparatively low overwintering mortality rate of 9.84% between 2009 and 2021 (Table 1) [58,59]. Compared with previous results, the calculated total colony losses (2009–2021) were higher than the losses reported in 2020 [28] and lower than those reported in 2016 [26] and 2017 [27]. A total loss of 12.8% was considered acceptable, and the loss rate in this survey was within the acceptable level [14]. In addition to differences in climate, management practices by Chinese beekeepers may explain the low mortality of colonies during winter. Based on the data (Figure 1C), 94% of the colonies were replaced with new queens at least once every year by 93% of beekeepers in this survey. With the high frequency and proportion of requeening by Chinese beekeepers, the stability and survival of honey bee colonies can be greatly improved by the emergence of young queens [60]. The health status of the queen can affect the development of existing colonies, including colony strength and pathogen resistance [61]. Increasing the proportion of young queens can improve colony health and increase the brood and oviposition numbers of the colonies [62].

The winter colony losses also varied among years, provinces, and operation sizes. Our results show that the annual losses in 2009–2010 and 2020–2021 were significantly different from those in other years. These differences may be caused by multiple factors, such as climate, precipitation, and nectar source, with the interacting effects of these factors. The minimum value, a 2.81% loss, was reported in 2009–2010 (our first year of the survey), and we suspect that inexperienced surveyors also affected the loss rate.

For provincial losses, different regions exhibited contrasting loss rates. The differences may be attributed to the landscapes, beekeeping practices, weather, and climate, and must be investigated in future [63,64,65]. In the present study, we received responses from Tibet for the first time, with only nine responses. Compared to other provinces, beekeeping in Tibet started late. The apiculture of Tibet is relatively backward, with a small number of managed honey bee colonies. The provincial loss of Gansu was always kept at a low level and was greatly associated with high-level beekeeping practices, which was referred to in previous surveys [26,28]. The colony loss in Gansu was significantly lower than that in the other provinces. The annual losses of six representative provinces demonstrated that the pattern of colony losses in one province remained stable during the surveyed years, which is also consistent with the findings of our previous studies [26,27]. Some provinces with low mortality, such as Gansu, tend to experience low mortality in all years in China. Such a pattern was not observed in Europe or the United States, where places with high/low colony losses varied among years [14,15,16,19,20,22]. The low mortality of Gansu may be due to its long apiculture history and excellent beekeeping skills [28].

In the present survey, most participants belonged to sideline beekeepers (69%; N = 9490), and the colonies of the hobby beekeepers only accounted for 5% of the colonies. Overall, the size of apiaries has increased in recent years across China but is still relatively small (Figure 4A). Although there were inconsistent phenomena in the annual losses of different operation sizes, the trend that a larger apiary had a lower loss can be observed, and the loss rate of commercial beekeepers was significantly lower than that of the other two types of beekeepers (Figure 4B). Colonies from large operations had a higher probability of overwinter survival, which has often been found in many other studies [23,38,66]. The following factors are likely to contribute to this trend: First, Chinese commercial beekeepers preferred to introduce good-quality queens from professional breeding institutions instead of breeding by themselves and 73% of beekeeper did this (Figure S2D). As a result, the performance of the whole population in apiaries could be adequately improved on a large scale [67,68,69]. Second, commercial beekeepers usually have excellent skills. More advanced beekeeping practices and anti-epidemic measures have been applied to commercial apiaries, reducing the risk of colony losses to some extent [11,28]. Further investigation into the management differences between small and large apiaries may help us find a way to reduce colony losses.

Considering that winter colony losses regarding *A. cerana* have been poorly studied, we first explored the differences in winter colony losses between *A. mellifera* and *A. cerana*. A long time ago, *A. mellifera* was introduced into China, and as a result, it has been tolerant to the climate of China and has become the dominant species in Chinese beekeeping [70,71]. Recently, many studies on the influence of alien honey bee species (*A. mellifera*) on local species (*A. cerana*) in food competition, disease transmission, and reproduction interference have been performed in China [72,73,74,75,76]. Meanwhile, the population size of *A. cerana* in China has decreased in recent years [46,51]. *A. cerana* may be even more threatened than *A. mellifera* in China. Our data showed that the total loss in *A. cerana* was significantly higher than that in *A. mellifera*. The annual losses of *A. mellifera* and *A. cerana* both differed from year to year. Regarding the loss rates of *A. mellifera* and *A. cerana* in different apiaries, colonies of *A. mellifera* with a large operation size had high losses, whereas colonies of *A. cerana* with a large operation size had low losses. These patterns were consistent with our previous results but contrasted with our previous study [28], which only covered a fraction of the data on *A. mellifera* honey bee colony losses used in the current study. Extending the survey to more years showed that the pattern of *A. mellifera* varied among years (Figure 5). The underlying reasons for the different patterns of colony winter loss between *A. mellifera* and *A. cerana* need to be studied further and identified. Additionally, beekeepers who migrated had significantly lower losses, and *A. mellifera* colonies used for pollination had significantly lower losses. Migration can increase the probability of colony overwinter survival, and pollination can increase the probability of colony overwinter survival [21,24], perhaps because these two operations contribute to acquiring sufficient and/or diverse food for honey bee colonies. Simone-Finstrom et al. showed that migrating colonies to agricultural areas with good nutrition can reduce oxidative stress in honey bees [77]. However, many studies have demonstrated that migratory pollination practices have varying health effects on honey bee colonies [78]. More research is necessary to explore the impact of migratory beekeeping on bee health, not only on pollinator health but also on food security [79,80]. As for the pollination, previous work found that the effect of pollination on honey bee health was heterogeneous, probably due to several uncontrolled underlying factors such as beekeeping management, species of pollinated crops, parasites, and pathogens [81,82,83]. It is a pity that detailed information about pollination was not included in our questionnaires, which limited our survey. In the future, we will make more of an effort to investigate pollination.

It was estimated by the GLMMs analysis that the significant risk factors were the operation size, species, migration, migration×species interaction, and queen problems. As mentioned previously, Chinese apiaries with large operation sizes showed significantly lower winter mortality. This is in accordance with the survey results of other countries [11,28,66]. Large operation sizes represent high-level beekeeping management practices and strong honey bee colonies, contributing to the overwintering of colonies. Many previous results have demonstrated that queen problems are significantly associated with winter colony losses, which was also observed in this survey [37,84,85]. A healthy queen was the most important factor affecting colony winter survival. A healthy queen can maintain the health status of the colonies and provide better control over swarming. Different honey bee species also had a significant influence on colony loss. Significantly lower losses were observed for *A. mellifera* in China. *A. mellifera* and *A. cerana* have some similarities but strongly differ in many aspects, such as their resistance to *V. destructor* [49,86], which may underlie the differences in colony survival [29]. Consistent with previous studies [21,23,66], our analysis revealed that migratory beekeepers experienced a lower loss rate. Comparing the percentage of migratory beekeepers with the percentage of beekeepers for pollination, we found that Chinese beekeepers migrated to their colonies mainly for honey harvest—not for pollination. Although migration increases the possibility of exposure to pathogens and pesticides, it also gives the colonies more access to better foraging sources. In addition, migratory beekeepers are often more well-trained and experienced. In many previous studies, migration was not always recognized as a significant factor in reducing colony losses [13]. However, this effect has been reported in several surveys [21,23,67]. Migration and species interaction also exists, possibly because *A. cerana* beekeeping is mainly stationary. It is well known that *V. destructor* can seriously lower the chance of colony survival, especially *A. mellifera*, and sometimes the damage is devastating [10,87,88]. However, treatment with *V. destructor* did not significantly correlate with colony loss in our GLMMs analysis. As shown in Figure S2A, 99% of Chinese beekeepers who managed *A. mellifera* treated their colonies against *Varroa* mites, and the number of colonies untreated in the survey may be insufficient to show the statistical significance of the effect of *Varroa* treatment on colony losses.

As for *A. cerana*, the Chinese COLOSS questionnaires were not designed specifically for *A. cerana*. Many questions about the unique traits of this species were not designed to be specific to our study. As a result, some important information may have been missed, and this something which we need to improve on in future surveys. Some questions about beekeeping styles (traditional or movable-frame hive), sacbrood disease, and honey yield could be included into our future COLOSS questionnaires specific to *A. cerana*.

## 5. Conclusions

Honey bee decline has been widely reported in recent years, raising concerns worldwide. Our China-based colony loss survey involved the highest number of colonies to date. Our survey showed the overall losses of managed honey bee colonies from 21 provinces in China between 2009 and 2021, and a relatively low mortality (below the world average level) was reported. Colony losses varied among years, provinces, and types of apiaries. Apiaries with larger operation sizes suffered lower colony losses. Additionally, to the best of our knowledge, this is the first national survey to reveal the differences in the pattern of winter colony losses between *A. mellifera* and *A. cerana*. Using GLMMs analysis, we explored the effects of risk factors on winter colony losses and found that queen problems, operation size, species, migration, and migration×species interaction were the primary risk factors that significantly affected the overwintering mortality of honey bees in China. Although treatment against *V. destructor* was not identified in our analysis because of insufficient data, *V. destructor* control is likely to be important for *A. mellifera* survival during winter. Future studies should increase response rates and include more refined information, and more efforts are needed to further investigate honey bee colony loss. The findings of this surveillance study provide more reliable and abundant insights into the health status of honey bee colonies in China and the pattern of winter colony losses in China.

## Figures and Tables

**Table 1:** Reported total colony losses of managed honey bees in China (2009–2021).

Year	No. of Apiaries	No. of Colonies	Total Losses % (95% CI)
2009–2021	13,704	1,744,324	9.84 (9.60–10.08)
2009–2010	1016	116,547	2.81 (2.38–3.32)
2010–2011	1586	188,580	9.65 (8.94–10.41)
2011–2012	1419	174,009	11.78 (10.74–12.91)
2012–2013	1509	176,171	9.97 (9.30–10.70)
2013–2014	1536	216,690	9.23 (8.49–10.03)
2014–2015	442	50,353	8.41 (7.32–9.64)
2015–2016	800	110,540	10.25 (9.20–11.39)
2016–2017	889	103,394	7.88 (7.29–8.52)
2017–2018	1279	162,849	9.82 (9.17–10.51)
2018–2019	668	80,427	11.17 (10.24–12.17)
2019–2020	821	98,591	10.49 (9.61–11.43)
2020–2021	1739	266,173	12.40 (11.76–13.06)

**Table 2:** Provincial total winter losses (%, CI 95%) and number of apiaries in China.

Province	No. of Apiaries	No. of Colonies	Total Losses % (95% CI)
Anhui	144	18,699	8.35 (7.36–9.47)
Beijing	174	17,352	11.19 (9.64–12.94)
Chongqing	800	93,459	9.43 (8.60–10.33)
Gansu	1134	87,483	2.09 (1.80–2.44)
Guangdong	1099	146,351	10.06 (9.38–10.79)
Guangxi	841	142,910	10.7 (8.5–12.8)
Guizhou	100	24,770	11.58 (8.57–15.48)
Hainan	574	46,293	15.67 (14.41–17.01)
Heilongjiang	312	31,352	7.86 (6.37–9.65)
Henan	870	70,543	16.34 (15.74–16.96)
Jiangsu	541	62,879	6.47 (5.82–7.19)
Jiangxi	337	41,263	16.25 (14.50–18.16)
Jilin	1188	109,019	8.12 (7.63–8.63)
Liaoning	996	91,527	7.80 (7.09–8.59)
Shandong	281	32,485	7.87 (6.70–9.22)
Shanxi	842	50,392	10.68 (9.70–11.75)
Sichuan	615	134,297	7.59 (6.71–8.58)
Xinjiang	878	272,405	15.22 (13.81–16.74)
Tibet ^1^	9	2,041	0.64 (0.03–14.07)
Yunnan	826	107,503	5.11 (4.48–5.82)
Zhejiang	1143	161,301	7.26 (6.53–8.07)

^1^ Tibet not included in the Kruskal–Wallis Test (sample size < 10).

**Table 3:** Total colony losses by different honey bee species in China (2009–2021).

Year	Species	No. of Apiaries	No. of Colonies	Total Losses % (95% CI)
2009–2021	*A. cerana*	4253	478,905	11.13 (10.73–11.55)
2009–2010	*A. mellifera* *A. cerana*	5711 330	847,587 30,973	9.38 (8.98–9.79) 1.56 (1.10–2.21)
2010–2011	*A. mellifera* *A. cerana*	345 446	56,575 39,779	2.06 (1.33–3.19) 9.85 (8.52–11.38)
2011–2012	*A. mellifera* *A. cerana*	545 463	86,287 39,625	10.02 (8.80–11.39)
				8.99 (7.79–10.36)
	*A. mellifera*	602	84,881	15.33 (13.41–17.48)
2012–2013	*A. cerana*	388	33,138	15.67 (14.11–17.37)
	*A. mellifera*	701	94,612	9.45 (8.45–10.55)
2013–2014	*A. cerana*	341	35,056	12.92 (11.54–14.43)
	*A. mellifera*	721	127,645	8.75 (7.57–10.09)
2014–2015	*A. cerana*	80	5554	12.69 (9.69–16.47)
	*A. mellifera*	75	12,976	4.08 (1.91–8.52)
2015–2016	*A. cerana*	207	14,981	16.89 (14.88–19.10)
	*A. mellifera*	452	71,759	10.14 (8.69–11.80)
2016–2017	*A. cerana*	308	37,090	8.75 (7.58–10.07)
	*A. mellifera*	282	32,229	5.32 (4.52–6.24)
2017–2018	*A. cerana*	391	56,232	9.82 (9.17–10.51)
	*A. mellifera*	660	85,578	8.28 (7.42–9.23)
2018–2019	*A. cerana*	320	40,435	12.83 (11.54–14.24)
	*A. mellifera*	198	24,231	9.46 (7.83–11.39)
2019–2020	*A. cerana*	304	43,774	9.17 (7.90–10.61)
	*A. mellifera*	276	34,023	9.53 (8.03–11.26)
2020–2021	*A. cerana*	675	102,268	14.02 (12.92–15.20)
	*A. mellifera*	854	136,791	10.56 (9.82–11.34)

**Table 4:** Risk factors for winter colony losses in the final model.

Risk Factors	Estimate (SE)	Z Value	*p*
Intercept	−3.313 (0.215)	−15.373	<2 × 10^−16^ ***
Operation Size (sideline)	0.754 (0.081)	9.335	<2 × 10^−16^ ***
Operation Size (hobby)	0.969 (0.009)	9.793	<2 × 10^−16^ ***
Species (*A. mellifera*)	−0.915 (0.084)	−10.902	<2 × 10^−16^ ***
Migration	−0.729 (0.090)	−8.068	7.17 × 10^−16^ ***
Queen Problem	0.111 (0.004)	24.678	<2 × 10^−16^ ***
Species (*A. mellifera*) × Migration	0.436 (0.108)	4.042	5.29 × 10^−5^ ***

SE = Standard error; Significant codes: 0.0001 ‘***’.

## Data Availability

The data presented in this study are available upon request from the corresponding authors. The data are not publicly available due to privacy concerns and potential political sensitivities.

## Supplementary Materials

The following supporting information can be downloaded at: https://www.mdpi.com/article/10.3390/insects14060554/s1, the file Supplementary Materials S1 (S1), Figure S1: The proportion of respondents/colonies surveyed in different years; Figure S2: (A) The proportion of respondents/colonies with *V. destructor* control or without *V. destructor* control. (B) Distribution of respondents/colonies with new combs, with every 10% of new combs divided into one group. (C) Proportion of migrated and pollinated respondents/colonies in the survey. (D) The proportion of respondents in different types of apiaries with three origins of queens; Table S1: Annual winter colony losses (%, CI: 95%) in Chongqing, Gansu, Guangdong, Guangxi, Jiangxi, and Zhejiang provinces of China (2017–2021). Figure S3: The proportion of respondents/colonies with queen problem in different types of apiaries. Supplementary Material S2 (S2) and GLMM model selection results.

## Abbreviations

COLOSS	prevention of honey bee COlony LOSSes.
FAO	the Food and Agriculture Organization of the United Nations
