# Supplementary material for: Survey Results of Honey Bee Colony Losses in Winter in China (2009–2021)
Source: Insects. 2023 Jun 14;14(6):554. doi: 10.3390/insects14060554 (PMC10299515; doi:10.3390/insects14060554)
Supplement: Supplementary file 1 [file insects-14-00554-s001.zip › Supplementary Materials S1-2297433.pdf]

Table S1. Annual winter colony losses (%, CI 95%) in Chongqing, Gansu, Guangdong, Guangxi, Jiangxi, and Zhejiang provinces of China (2017–2021).

Province	Year			
	2017–2018	2018–2019	2019–2020	2020–2021
Chongqing	9.35(7.59–11.47)	10.01(8.54–11.71)	15.17 (12.22–18.91)	15.92(12.99–19.37)
Gansu	1.48(0.98–2.22)	3.31(2.41–4.52)	1.08 (0.65–1.78)	2.34(1.66–3.31)
Guangdong	11.97(10.44–13.69)	11.88(10.19–13.81)	12.28 (10.38–14.47)	17.84(15.35–20.66)
Guangxi	7.52(5.42–10.36)	11.71(9.09–14.97)	8.81 (6.91–11.17)	13.33(11.08–15.94)
Jiangxi	26.18(21.87–31.00)	26.56(23.17–30.24)	9.74 (6.22–14.93)	12.74(9.86–16.31)
Zhejiang	8.04(5.71–11.21)	9.71(7.64–12.28)	9.37 (7.27–11.99)	10.87(9.10–12.94)

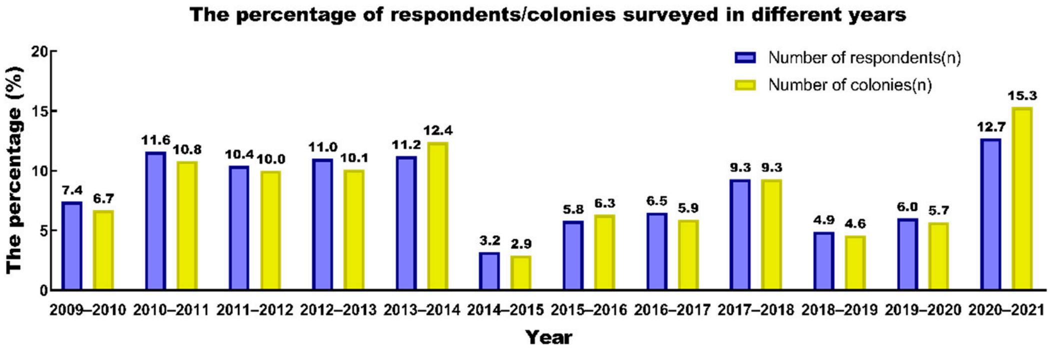

Figure S1. The proportion of respondents/colonies surveyed in different years.

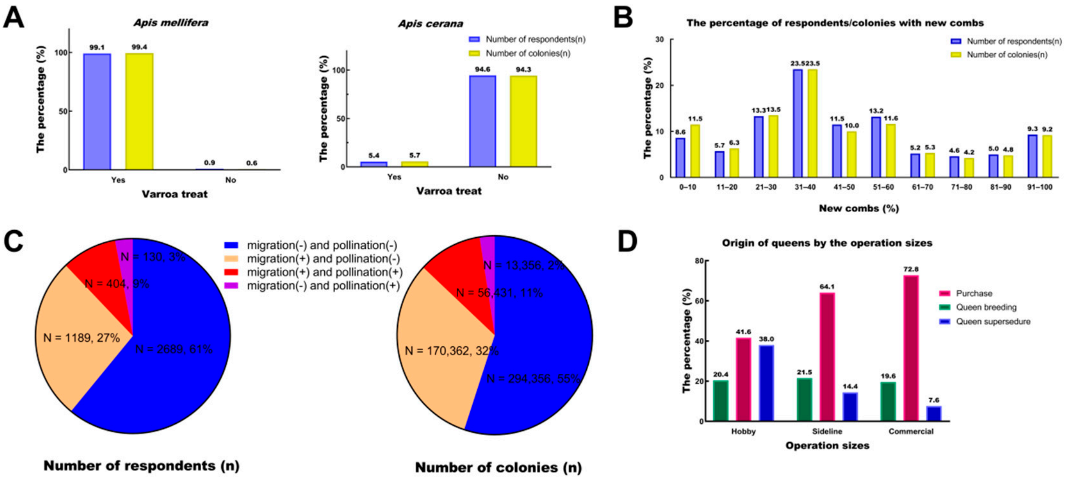

Figure S2. The proportion of respondents/colonies with different treatments. (A) The proportion of respondents/colonies with *V. destructor* control or without *V. destructor* control. (B) The distribution of respondents/colonies with new combs, with every 10% of new comb divided into one group. (C) The proportion of migrated and pollinated respondents/colonies in the survey. (D) The proportion of respondents in different types of apiaries with three origin of queen.

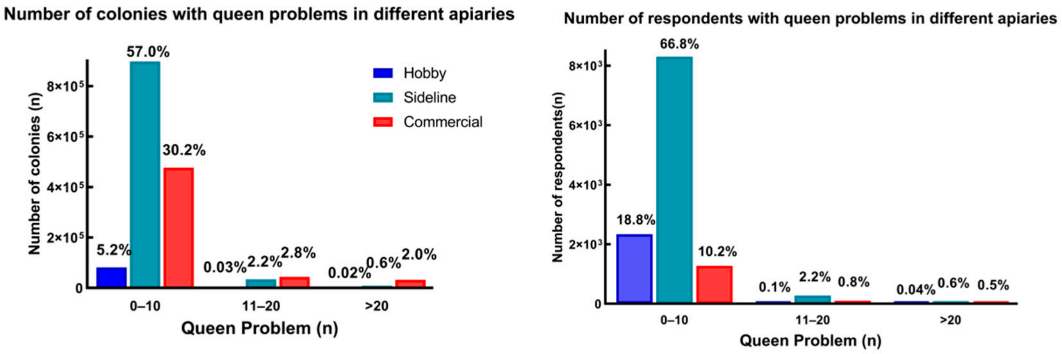

Figure S3. The proportion of respondents/colonies with queen problem in different types of apiaries.
