# Supplementary material for: Survey Results of Honey Bee Colony Losses in Winter in China (2009–2021)
Source: Insects. 2023 Jun 14;14(6):554. doi: 10.3390/insects14060554 (PMC10299515; doi:10.3390/insects14060554)
Supplement: Supplementary file 1 [file insects-14-00554-s001.zip › Supplementary Materials S2.pdf]

Table S2. Summary results of GLMM models fitting .

Models with Fixed Effects	AIC	BIC
Size, species, migration, qp	31224.85422	31283.29033
Size, species, vt, migration, qp	31225.65337	31290.58237
Size, species, Requeening, migration, qp	31226.16104	31291.09005
Size, species, migration, qp, wf	31226.65206	31291.58106
Size, vt, migration, qp	31233.9127	31292.34881
Size, species, oq, migration, qp	31222.98461	31294.40651
Size, species, Requeening, vt, migration, qp	31226.84923	31298.27113
Size, species, vt, migration, qp, wf	31227.50892	31298.93083
Size, species, Requeening, migration, qp, wf	31227.79271	31299.21462
Size, Requeening, vt, migration, qp	31235.55357	31300.48257
Size, vt, migration, qp, wf	31235.78314	31300.71214
Size, oq, vt, migration, qp	31229.93517	31301.35707
Size, species, oq, vt, migration, qp	31223.57926	31301.49407
Size, species, oq, migration, qp, wf	31223.94583	31301.86063
Size, species, Requeening, oq, migration, qp	31224.77612	31302.69093
Size, species, Requeening, vt, migration, qp, wf	31228.55252	31306.46732
Size, Requeening, vt, migration, qp, wf	31237.33199	31308.7539
Size, oq, vt, migration, qp, wf	31230.97519	31308.88999
Size, species, oq, vt, migration, qp, wf	31224.65559	31309.0633
Size, species, Requeening, oq, vt, migration, qp	31225.33461	31309.74232
Size, Requeening, oq, vt, migration, qp	31231.86378	31309.77858
Size, species, Requeening, oq, migration, qp, wf	31225.59293	31310.00064
Size, species, Requeening, oq, vt, migration, qp, wf	31226.26706	31317.16767
Size, Requeening, oq, vt, migration, qp, wf	31232.81807	31317.22578
Size, oq, migration, qp	31268.65983	31333.58883
species, migration, qp	31292.81412	31338.26443
Size, oq, migration, qp, wf	31268.07965	31339.50156
Size, migration, qp	31287.90337	31339.84657
Size, Requeening, oq, migration, qp	31269.98353	31341.40543
species, vt, migration, qp	31293.3228	31345.266
species, migration, qp, wf	31294.35333	31346.29653
Size, Requeening, migration, qp	31288.1234	31346.55951
species, Requeening, migration, qp	31294.75713	31346.70034
Size, migration, qp, wf	31288.42935	31346.86545
Size, species, qp	31295.14026	31347.08346
vt, migration, qp	31301.85957	31347.30987
Size, Requeening, oq, migration, qp, wf	31269.72175	31347.63656
species, oq, migration, qp	31290.85994	31349.29604
species, vt, migration, qp, wf	31294.96121	31353.39731
species, Requeening, vt, migration, qp	31295.22617	31353.66227

Size, Requeening, migration, qp, wf	31289.17963	31354.10863
Size, species, vt, qp	31295.79738	31354.23348
species, Requeening, migration, qp, wf	31296.21855	31354.65465
Size, species, qp, wf	31296.67053	31355.10663
Size, species, Requeening, qp	31296.9774	31355.4135
vt, migration, qp, wf	31303.52313	31355.46633
Requeening, vt, migration, qp	31303.86261	31355.80581
oq, vt, migration, qp	31297.47652	31355.91262
species, oq, vt, migration, qp	31291.28355	31356.21255
species, oq, migration, qp, wf	31291.4422	31356.3712
Size, species, oq, qp	31292.54584	31357.47484
species, Requeening, oq, migration, qp	31292.86234	31357.79135
species, Requeening, vt, migration, qp, wf	31296.7819	31361.71091
Size, species, vt, qp, wf	31297.42305	31362.35205
Size, species, Requeening, vt, qp	31297.57541	31362.50441
oq, vt, migration, qp, wf	31298.16993	31363.09894
Size, species, Requeening, qp, wf	31298.38484	31363.31385
species, oq, vt, migration, qp, wf	31292.01005	31363.43196
Requeening, vt, migration, qp, wf	31305.51468	31363.95079
Requeening, oq, vt, migration, qp	31299.43479	31364.3638
Size, species, oq, qp, wf	31292.99221	31364.41412
Size, species, oq, vt, qp	31293.07437	31364.49628
species, Requeening, oq, vt, migration, qp	31293.28618	31364.70809
species, Requeening, oq, migration, qp, wf	31293.42787	31364.84977
Size, vt, qp	31313.37762	31365.32082
Size, species, Requeening, oq, qp	31294.53632	31365.95823
Size, species, Requeening, vt, qp, wf	31299.07779	31370.4997
Size, species, oq, vt, qp, wf	31293.66527	31371.58008
Requeening, oq, vt, migration, qp, wf	31300.16891	31371.59082
species, Requeening, oq, vt, migration, qp, wf	31293.98681	31371.90162
Size, oq, vt, qp	31307.0801	31372.00911
Size, species, Requeening, oq, qp, wf	31294.92324	31372.83805
Size, species, Requeening, oq, vt, qp	31295.05575	31372.97056
Size, vt, qp, wf	31315.01716	31373.45327
Size, Requeening, vt, qp	31315.37682	31373.81292
Size, oq, vt, qp, wf	31307.57653	31378.99844
Size, species, Requeening, oq, vt, qp, wf	31295.57981	31379.98752
Size, Requeening, oq, vt, qp	31309.03969	31380.4616
Size, Requeening, vt, qp, wf	31316.99856	31381.92756
Size, Requeening, oq, vt, qp, wf	31309.57487	31387.48968
oq, migration, qp	31337.31471	31389.25792
oq, migration, qp, wf	31336.17283	31394.60894
Requeening, oq, migration, qp	31337.56735	31396.00345

migration, qp	31361.04833	31400.00574
Requeening, oq, migration, qp, wf	31337.0056	31401.9346
Requeening, migration, qp	31358.8694	31404.31971
migration, qp, wf	31360.87059	31406.3209
Requeening, migration, qp, wf	31359.66976	31411.61296
species, qp	31389.96974	31428.92714
Size, oq, qp	31373.7977	31432.2338
species, vt, qp	31390.24963	31435.69993
Size, oq, qp, wf	31371.36991	31436.29891
species, qp, wf	31390.99021	31436.44052
species, Requeening, qp	31391.74104	31437.19134
Size, Requeening, oq, qp	31372.86109	31437.7901
species, oq, qp	31386.6221	31438.5653
Size, Requeening, oq, qp, wf	31371.35221	31442.77412
species, vt, qp, wf	31391.4278	31443.371
species, Requeening, vt, qp	31392.0885	31444.0317
species, Requeening, qp, wf	31392.8986	31444.8418
species, oq, qp, wf	31386.54037	31444.97648
species, oq, vt, qp	31386.98695	31445.42305
species, Requeening, oq, qp	31388.27469	31446.71079
vt, qp	31411.06527	31450.02267
species, Requeening, vt, qp, wf	31393.36948	31451.80558
species, oq, vt, qp, wf	31387.08318	31452.01218
species, Requeening, oq, qp, wf	31388.37821	31453.30721
species, Requeening, oq, vt, qp	31388.68598	31453.61498
oq, vt, qp	31402.89024	31454.83345
Requeening, vt, qp	31412.23724	31457.68754
vt, qp, wf	31412.26114	31457.71144
Size, qp	31412.52265	31457.97295
Size, Requeening, qp	31406.94295	31458.88615
species, Requeening, oq, vt, qp, wf	31388.94519	31460.3671
oq, vt, qp, wf	31402.88716	31461.32326
Requeening, oq, vt, qp	31404.0406	31462.4767
Size, qp, wf	31411.05394	31462.99714
Size, Requeening, qp, wf	31407.16286	31465.59897
Requeening, vt, qp, wf	31413.67719	31465.6204
Requeening, oq, vt, qp, wf	31404.33301	31469.26201
oq, qp	31478.17533	31523.62563
Requeening, oq, qp	31473.56659	31525.50979
oq, qp, wf	31474.69896	31526.64216
Requeening, oq, qp, wf	31471.62695	31530.06305
Requeening, qp	31517.38659	31556.34399
Requeening, qp, wf	31517.10013	31562.55043

qp	31532.26732	31564.73183
qp, wf	31528.87457	31567.83197
Size, species, migration	31517.84806	31569.79126
Size, species, vt, migration	31518.27017	31576.70628
Size, species, migration, wf	31518.91861	31577.35471
Size, species, Requeening, migration	31519.58635	31578.02245
Size, vt, migration	31528.09189	31580.03509
Size, species, oq, migration	31519.05219	31583.9812
Size, species, vt, migration, wf	31519.48763	31584.41663
Size, species, Requeening, vt, migration	31519.93042	31584.85943
Size, species, Requeening, migration, wf	31520.43715	31585.36615
Size, vt, migration, wf	31529.34657	31587.78268
Size, Requeening, vt, migration	31530.02354	31588.45965
Size, species, oq, migration, wf	31519.39561	31590.81751
Size, species, oq, vt, migration	31519.48251	31590.90442
Size, oq, vt, migration	31527.20772	31592.13672
Size, species, Requeening, oq, migration	31520.86899	31592.29089
Size, species, Requeening, vt, migration, wf	31520.92682	31592.34872
Size, Requeening, vt, migration, wf	31531.1723	31596.10131
Size, species, oq, vt, migration, wf	31519.98118	31597.89599
Size, species, Requeening, oq, migration, wf	31521.0356	31598.95041
Size, oq, vt, migration, wf	31527.65858	31599.08048
Size, species, Requeening, oq, vt, migration	31521.26477	31599.17958
Size, Requeening, oq, vt, migration	31529.15928	31600.58119
Size, species, Requeening, oq, vt, migration, wf	31521.58543	31605.99313
Size, Requeening, oq, vt, migration, wf	31529.51184	31607.42665
Size, oq, migration	31571.86079	31630.29689
Size, oq, migration, wf	31570.19527	31635.12428
Size, species	31590.24681	31635.69711
Size, Requeening, oq, migration	31572.98101	31637.91002
Size, migration	31593.36865	31638.81896
Size, species, vt	31590.49875	31642.44195
Size, species, wf	31590.81331	31642.75651
Size, Requeening, oq, migration, wf	31571.74571	31643.16762
Size, Requeening, migration	31591.94084	31643.88404
Size, migration, wf	31592.11665	31644.05985
Size, species, Requeening	31592.24164	31644.18484
Size, species, oq	31590.34207	31648.77817
Size, species, vt, wf	31591.25877	31649.69487
Size, Requeening, migration, wf	31591.78624	31650.22235
Size, species, Requeening, vt	31592.47697	31650.91308
Size, species, Requeening, wf	31592.72821	31651.16431
Size, species, oq, wf	31590.06252	31654.99153

Size, species, oq, vt	31590.69693	31655.62593
Size, vt	31610.43808	31655.88838
Size, species, Requeening, oq	31592.33852	31657.26752
Size, species, Requeening, vt, wf	31593.137	31658.06601
species, migration	31620.52716	31659.48456
Size, species, oq, vt, wf	31590.60429	31662.0262
Size, vt, wf	31611.22469	31663.16789
Size, species, Requeening, oq, wf	31591.99508	31663.41698
Size, species, Requeening, oq, vt	31592.68698	31664.10889
Size, Requeening, vt	31612.33526	31664.27846
Size, oq, vt	31606.72591	31665.16201
species, vt, migration	31620.56229	31666.01259
species, migration, wf	31620.96893	31666.41923
species, Requeening, migration	31622.37032	31667.82062
vt, migration	31631.47507	31670.43247
Size, species, Requeening, oq, vt, wf	31592.52063	31670.43544
Size, oq, vt, wf	31606.51513	31671.44414
Size, Requeening, vt, wf	31613.20783	31671.64394
species, oq, migration	31620.56767	31672.51087
species, vt, migration, wf	31621.21744	31673.16065
Size, Requeening, oq, vt	31608.65946	31673.58847
species, Requeening, vt, migration	31622.46252	31674.40572
species, Requeening, migration, wf	31622.94015	31674.88336
vt, migration, wf	31632.18142	31677.63173
Requeening, vt, migration	31633.0333	31678.4836
species, oq, migration, wf	31620.52428	31678.96039
species, oq, vt, migration	31620.86555	31679.30165
Size, Requeening, oq, vt, wf	31608.51114	31679.93304
oq, vt, migration	31628.90469	31680.8479
species, Requeening, oq, migration	31622.50633	31680.94243
species, Requeening, vt, migration, wf	31623.2071	31681.6432
Requeening, vt, migration, wf	31633.95787	31685.90107
species, oq, vt, migration, wf	31621.00354	31685.93254
oq, vt, migration, wf	31628.99999	31687.43609
species, Requeening, oq, migration, wf	31622.52336	31687.45237
species, Requeening, oq, vt, migration	31622.82292	31687.75192
Requeening, oq, vt, migration	31630.68736	31689.12347
species, Requeening, oq, vt, migration, wf	31623.00607	31694.42798
Requeening, oq, vt, migration, wf	31630.91634	31695.84535
oq, migration	31675.98811	31721.43841
oq, migration, wf	31673.73721	31725.68041
Requeening, oq, migration	31675.21127	31727.15448
Requeening, oq, migration, wf	31673.81493	31732.25104

Size, oq	31681.88028	31733.82349
Size, oq, wf	31678.04597	31736.48207
Requeening, migration	31699.86814	31738.82554
Size, Requeening, oq	31680.45065	31738.88676
migration	31706.66235	31739.12685
Size, Requeening, oq, wf	31677.75163	31742.68064
migration, wf	31704.03484	31742.99225
Requeening, migration, wf	31699.32257	31744.77288
Size, Requeening	31717.472	31762.9223
species	31732.98887	31765.45337
Size	31726.55228	31765.50969
Size, wf	31722.461	31767.9113
Size, Requeening, wf	31716.11317	31768.05638
species, wf	31732.47182	31771.42923
species, vt	31732.71817	31771.67557
species, Requeening	31733.55365	31772.51106
species, oq	31729.89409	31775.34439
species, vt, wf	31732.49462	31777.94492
species, Requeening, vt	31733.47887	31778.92918
species, Requeening, wf	31733.61333	31779.06364
species, oq, wf	31729.02913	31780.97234
species, oq, vt	31730.11531	31782.05852
species, Requeening, oq	31731.13778	31783.08098
species, Requeening, vt, wf	31733.75165	31785.69486
species, oq, vt, wf	31729.46979	31787.90589
species, Requeening, oq, wf	31730.60177	31789.03787
species, Requeening, oq, vt	31731.42803	31789.86414
vt	31758.32668	31790.79119
oq, vt	31749.65286	31795.10317
species, Requeening, oq, vt, wf	31731.08113	31796.01014
Requeening, vt	31757.52455	31796.48195
vt, wf	31758.12768	31797.08509
oq, vt, wf	31748.8815	31800.8247
Requeening, oq, vt	31750.11663	31802.05984
Requeening, vt, wf	31758.06821	31803.51852
Requeening, oq, vt, wf	31749.81488	31808.25099
Requeening, oq	31831.26205	31876.71236
oq	31838.38699	31877.3444
oq, wf	31833.27376	31878.72407
Requeening, oq, wf	31828.20585	31880.14905
Requeening	31884.68209	31917.1466
Requeening, wf	31882.4847	31921.4421
wf	31902.86484	31935.32934

none	31910.4242	31936.3958

Size = operation size; Requeening = frequency of requeening; oq = origin of queen; vt = treatment against varroa; qp = queen problems; wf = winter food.

AIC = Akaike Information Criterion

BIC = Bayesian Information Criterion

All of the models include year, province and beekeeper as random effects.
